# Chronic insertional Achilles tendinopathy secondary to congenital os Achilles: A case report

**DOI:** 10.1016/j.ijscr.2022.107355

**Published:** 2022-06-27

**Authors:** Frederic J. Washburn, Emerald Chiang, Casey Pyle

**Affiliations:** a Community Memorial Health System, Orthopaedic Surgery Residency Program, 147 N. Brent Street, Ventura, CA 93003, USA; b Western University of Health Sciences College of Osteopathic Medicine of the Pacific, 309 E. Second Street, Pomona, CA 91766, USA

**Keywords:** Achilles tendinopathy, Haglund deformity, Ossification center, Case report

## Abstract

**Introduction:**

Insertional Achilles tendinopathy is a common overuse disorder affecting the foot and ankle that can lead to the development of a Haglund's deformity with chronicity, a retrocalcaneal exostosis that forms at the Achilles insertion site, further increasing pain and dysfunction.

**Presentation of case:**

We report a case of a healthy, 35–40-year-old male with chronic left-sided insertional Achilles pain beginning in early adolescence. Physical exam demonstrated bilateral prominences on the posterior aspect of both heels, exquisitely tender on the left and without range of motion deficits. Imaging demonstrated a large calcific ossicle clearly within the tendinous insertion of the Achilles onto the left calcaneus. He underwent surgical intervention to provide pain relief and restore function. He exhibited full recovery post-operatively and has now returned to full functional activities.

**Discussion:**

Given his symptom pathogenesis and progression, this patient may likely have suffered from chronic insertional Achilles tendinopathy due to an accessory ossicle that we believe was congenital. Current literature describes an additional secondary ossification center that appears over the dorsal, posterosuperior surface of the calcaneus. We suspect that there was a lapse in fusion at this additional ossification center that contributed to his pathological condition.

**Conclusion:**

This case report presents a unique occurrence of Achilles tendinopathy likely due to an accessory ossicle of congenital etiology. This highlights the importance of investigating the prevalence of this condition in those with chronic insertional Achilles tendinopathy, thus providing meaningful insight in considering effective treatment modalities in the management of these patients.

## Highlights

• Insertional Achilles tendinopathy is multifactorial, but mostly an overuse disorder.
• This is a unique case of a successfully treated IAT due to congenital os Achilles.
• Consideration of congenital os Achilles is valuable in evaluating chronic IAT.
• More studies will help guide future recognition and management for this condition.

## 1 Introduction

Insertional Achilles tendinopathy (IAT) is a common overuse disorder affecting the foot and ankle [1]. IAT mostly affects sedentary, middle-aged individuals with gastrocnemius or Achilles contractures but occurs in the younger population as well, with associated retrocalcaneal bursitis and Haglund deformity [1]. The exact pathophysiology of IAT is unclear, although thought to be multifactorial involving overuse, mechanical irritation, and bursitis contributing to microtrauma over time [1]. Patients typically present with posterior heel pain and swelling that worsens with physical activity, along with stiffness at rest [2]. Chronic insertional Achilles tendinopathy is initially treated with a trial of conservative management, which may include immobilization, activity modification, orthotics, and physical therapy with surgical intervention considered in refractory cases [3].

We present a unique case of a 35–40-year-old male with chronic left-sided insertional Achilles tendinopathy secondary to an accessory ossicle within the Achilles tendon. Typically, the calcaneal ossification process involves two sites that fuse prenatally with formation of an apophysis that fuses in adolescence [4]. A study performed by Nicholson et al. reports findings of an additional secondary ossification site along the posterosuperior portion of the calcaneus [5]. Therefore, we believe this ossicle to be congenital in nature as opposed to developing through the typical chronic disease process. This case report highlights our rationale for this atypical finding and describes our operative technique utilized to treat this unusual symptomatic os Achilles. The patient was managed in a community hospital setting and this case report was designed in-line with the Surgical Case Report 2020 Guidelines [6].

## 2 Case presentation

**Fig. 1:**
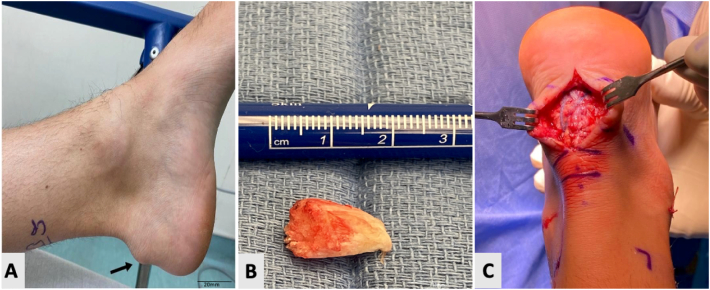
(A) Preoperative clinical image of the left ankle demonstrating a bony prominence at the posterosuperior aspect of the calcaneus (black arrow). (B) Clinical image demonstrating resected ossicle, measuring approximately 20 mm × 12 mm × 8 mm in size. (C) Intraoperative image taken after a central tendon splitting approach was used to detach and reattach the Achilles tendon using the SutureBridge double-row technique with SwiveLock anchors (Arthrex, Naples, FL).

Our patient is a healthy, 35–40-year-old male who presented to our clinic in Ventura, California for evaluation of chronic left-sided insertional Achilles pain with no history of trauma. His symptoms began in early adolescence, which limited his participation in competitive sports. He underwent multiple bouts of conservative management, with some periods of respite, but the severity of his pain progressively worsened over time and did not improve with skeletal maturity. On initial presentation to our clinic, he was unable to participate in any form of physical activity and footwear was limited to sandals as any pressure on his posterior heel caused severe pain. Physical examination was notable for a large prominence on the posterior aspect of both heels (Fig. 1A). He experienced exquisite tenderness to palpation of the left posterior prominence. Both feet exhibited full range of motion in dorsiflexion and no weakness in plantar flexion. He had a negative Silfverskiold test. The right sided prominence was asymptomatic.

**Fig. 2:**
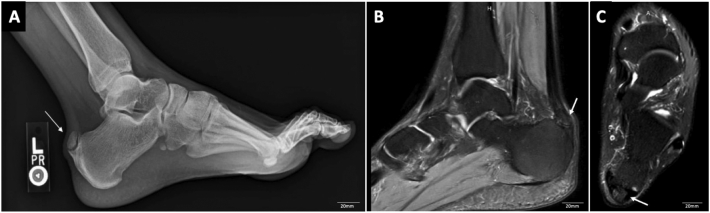
(A) Weightbearing lateral radiograph of the left ankle demonstrating presence of a large calcific ossicle at the posterosuperior aspect of the calcaneus (white arrow). Sagittal (2B) and axial (2C) T2-weighted magnetic resonance imaging of the left ankle revealing an ossicle at the posterosuperior aspect of the calcaneus in the tendinous insertion of the Achilles' tendon.

Left ankle weight-bearing radiographs demonstrated a large calcific ossicle within the tendinous insertion of the Achilles onto the calcaneus (Fig. 2A). Interestingly on MRI, the tendon itself exhibited minimal tendinosis with no signs of tendon degeneration. However, there was inflammation at the fibrous site between the ossicle and the calcaneus itself (Fig. 2B and C). Given these findings and the fact that the patient had failed extensive conservative management, surgical intervention in the form of excision of the ossicle itself, debridement, secondary repair of the Achilles tendon, and partial calcanectomy to reshape and contour the bone was planned to provide pain relief, improve function, and allow tendinous healing. The surgical procedure was performed by a foot and ankle fellowship-trained orthopaedic surgeon with assistance from an orthopaedic surgery resident.

## 3 Surgical intervention

The patient was placed in a prone position on the operating room table and a nonsterile tourniquet was placed around the left thigh. After preparing the operative extremity in the usual sterile fashion, fluoroscopic imaging was utilized to verify position of the ossicle for incision planning. A midline incision was made by straight dissection from skin to the ossicle through the Achilles tendon and a subperiosteal flap along the ossicle itself was created. The Achilles tendon was directly and carefully peeled off the ossicle, skeletonizing the fragment. The resected ossicle measured approximately 20 mm × 12 mm × 8 mm in size (Fig. 1B).

**Fig. 3:**
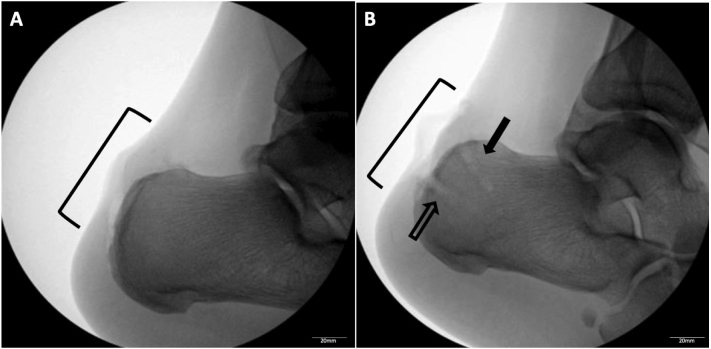
(A) Intraoperative lateral fluoroscopic image of the left calcaneus after excision of the ossicle, debridement, and partial calcanectomy in the region shown (black bracket). (B) Intraoperative lateral fluoroscopic image of the left calcaneus demonstrating proximal and distal anchor sites prior to insertion of the SwiveLock anchors (Arthrex, Naples, FL). After resection of the distal Achilles tendon, four anchor sites were marked. Two proximal (solid black arrow) and two distal (empty black arrow) anchor sites were drilled and tapped. Fluoroscopy was utilized to visualize proper anchor positioning.

The Achilles tendon was then elevated off its insertion on the calcaneus. The medial and lateral margins of the tendon were preserved to help maintain tendon length and tension. The calcaneal tuberosity was then rasped and contoured to remove any further bony prominence for preparation of the bone bed in receiving the tendon repair (Fig. 3A). The tendon was investigated, and a small portion of distal tendon was determined to be tendinotic and was resected. Two proximal anchor sites at the upper margin of the calcaneus were drilled and tapped prior to insertion of the anchors. This process was repeated distally to achieve the maximum Achilles footprint (Fig. 3B).

Two rescue sutures from the proximal anchors were stitched through the Achilles tendon proximal to the midline incision in horizontal mattress fashion. Double loaded suture tapes were then passed just distal to this. The proximal horizontal mattress sutures were tied down in a “spot weld” fashion to help secure the tendon to the bone and to set resting tension. The double loaded suture tapes were crossed and loaded into SwiveLock anchors (Arthrex, Naples, FL). Prior to tying these all down, a 0-Vicryl running suture was run through the midline incision of the Achilles tendon to help approximate the two margins together to restore the anatomy of the Achilles tendon. After this, the two distal holes for the anchors were localized and the loaded SwiveLock anchors (Arthrex, Naples, FL) were inserted in this distal row, thus completing our double row repair (Fig. 1C). Tension was set by maintaining the ankle in plantarflexion when tying the sutures and passing anchors. The resting tension held nicely after completion of the repair. All suture tapes and tails were cut, and anchor positioning was verified both clinically and under fluoroscopy. There was a negative Thompson test, and the repair was found to be secure. After irrigation, closure, and sterile dressing placement, a well-padded short leg splint was applied in resting plantar flexion. Post-operatively, the patient was kept non-weightbearing for two weeks, and then progressed to weightbearing as tolerated in a Controlled Ankle Movement Boot with heel wedge for an additional four weeks.

## 4 Discussion

The calcaneus is the first of the tarsal bones to undergo ossification and is known to begin generally around the 22 to 25th week of fetal development [4]. The process begins with two ossification centers that fuse prenatally in addition to an apophysis that forms along the posteroinferior aspect of the calcaneus which fuses by age 12–15 [4]. As an insertion site for muscular and tendinous structures, apophyses can be more prone to acute and chronic injuries in active children, especially during peak height velocity, due to its weaker nature in comparison to surrounding tendons and ligaments [7]. The calcaneal apophysis is implicated in calcaneal apophysitis (Sever's disease) in which mechanical overuse leads to a disruption in a system of diverging forces created by the traction of the Achilles tendon and plantar aponeurosis [4]. Similar examples can be seen in conditions such as Osgood-Schlatter disease, in which repetitive strain on the secondary ossification center of the tibial tubercle results in an apophysitis [8].

In this case, the patient's symptoms began as an early adolescent when he started participating in recreational sports. Physical examination revealed an identical, though asymptomatic ossicle on the contralateral foot that had similar time in onset. Intra-operatively, there was minimal Achilles tendinosis present, which is extremely unusual given the duration and severity of our patient's symptoms. Given vulnerability at apophyses during ossification, we suspect a lapse in fusion resulting in an additional ossification center that pathologically contributed to the development of an exostosis, similar to a Haglund deformity. Nicholson et al. showed that during later stages of ossification, 46 % of their cohort developed an additional secondary ossification center that appeared over the dorsal, posterosuperior surface of the calcaneus, supporting our suspicion [5]. Typical ossification of the calcaneal apophysis involves plantar extension before excursion over the dorsal region [5]. Of consideration is this interval between the metaphysis and apophysis during ossification; it is wider near regions of extension versus in the central region [5]. The implications of this morphology is unclear, but this non-uniform nature of ossification could potentially create an area predisposed to pathology and may be of consideration in determining the pathogenesis of an additional secondary ossification center. To our knowledge, the study performed by Nicholson et al. is the only documentation of this phenomenon occurring in current literature and this case report demonstrates an importance in investigating potential additional ossification centers likely contributing to pathological chronic insertional Achilles tendonitis [5].

Surgical intervention in the treatment of chronic insertional Achilles tendinitis resulting in a Haglund deformity is commonly recommended after failing 3 to 6 months of nonoperative treatment modalities such as activity modification, medications, or orthotic devices [9]. To treat this patient's condition, our surgical technique was similar to treating Haglund's deformity operatively. A central tendon splitting approach was used with SutureBridge (Arthrex, Naples, FL) double-row technique to excise the posterosuperior calcaneal ossicle as well as debride and reattach the Achilles tendon. In a systematic review by Thompson et al., all four detachment approaches (partial lateral or medial, complete, or tendon splitting approach) revealed similar rates of infection, postoperative rupture, wound complications, and future revision [10]. Another systematic review demonstrated that in general, surgical correction of Haglund's deformity provided good clinical outcomes as well as high patient satisfaction [11]. In addition, endoscopic procedures provided a lower rate of complications and failures, with shorter recovery time when compared to other operative modalities [11].

Our patient is now over six months post-surgery, and he is pain free at the Achilles insertional site. He has returned fully to his functional activities including jogging and is able to wear regular sneakers without experiencing discomfort. There have been no post-operative complications to date.

## 5 Conclusion

In conclusion, this case report is a unique occurrence of insertional Achilles tendinopathy likely due to a congenital accessory ossicle in contrast to the typical disease process of IAT. This case report highlights the importance of investigating this interesting pathology and the necessity of increased reported literature of this condition amongst those with chronic insertional Achilles tendinopathy. The presence of this accessory ossicle may be a more common occurrence than expected and any meaningful insight into possible physiologic mechanisms would benefit effective treatment modalities for this patient population, including early intervention.

## Consent

Written informed consent was obtained from the patient for publication of this case report and accompanying images. A copy of the written consent is available for review by the Editor-in-Chief of this journal upon request.

## Provenance and peer review

Not commissioned, externally peer-reviewed.

## Ethical approval

This case report was written with the approval of the CMHS Institutional Review Board.

## Funding

None. This research did not receive any specific grant from funding agencies in the public, commercial, or not-for-profit sectors.

## Guarantor

Frederic J. Washburn, DO.

## Research registration number

Not applicable.

## CRediT authorship contribution statement

All authors contributed equally to the composition of this manuscript.

## Declaration of competing interest

The authors declare that there are no conflicts of interest regarding the publication of this paper.
